# Non-linear relationship between triglyceride glucose-body mass index and risk of diabetes in adults: a general population-based cohort study of Chinese adults using a publicly available DRYAD dataset

**DOI:** 10.3389/fendo.2026.1823392

**Published:** 2026-05-25

**Authors:** Shanshan Xiao, Chunyan Du, Nini Zhang, Xun Jiang, Tianxin Hu

**Affiliations:** 1 Department of Pediatrics, Tangdu Hospital, Fourth Military Medical University, Xi’an, Shaanxi, China; 2 Department of Pharmacy, Tangdu Hospital, Fourth Military Medical University, Xi’an, Shaanxi, China

**Keywords:** Chinese adults, diabetes, non-linear association, threshold effect, TyG-BMI

## Abstract

**Objective:**

Despite triglyceride glucose-body mass index (TyG-BMI) being a validated marker of insulin resistance, its prospective association with diabetes risk remains unclear; we therefore examined the impact of TyG-BMI on incident diabetes.

**Methods:**

This was a retrospective cohort study based on 48,288 adults without diabetes at baseline, identified from a publicly available dataset on the DRYAD platform originally derived from 11 cities and 32 locations in China.Using Cox proportional risk regression modeling combined with cubic spline functions and smoothed curve fitting, we assessed the relationship between the baseline TyG-BMI and the risk of developing DM and explored its nonlinear association. We performed subgroup analyses to assess the consistency of the association across different subgroups.

**Results:**

Among 48,288 initially diabetes mellitus (DM)-free participants, 1,230 (2.54%) developed diabetes at follow-up.Adjusted for covariates, TyG-BMI levels(per 10-unit) were positively associated with the risk of diabetes onset (HR = 1.25,95% CI: 1.20-1.29, *P* < 0.001). The risk of incident diabetes increased progressively across quartiles of TyG-BMI levels (Q1 to Q4), with a significantly higher risk in Q4 compared to Q1 (adjusted HR = 11.24,95% CI = 5.05-24.99).Furthermore, a threshold effect of TyG-BMI on DM risk was found, with a threshold of 163.38. The HR to the right of the inflection point was 1.025 (95% CI: 1.020-1.029). When TyG-BMI was <163.38, the association was not statistically significant(HR = 0.928; 95% CI: 0.843–1.022), suggesting that the relationship may be absent or even slightly inverse.

**Conclusion:**

In Chinese adults, TyG-BMI exhibits a threshold effect on incident diabetes. Individuals above the threshold have a significantly increased risk of developing diabetes. However, the identified threshold (163.38) requires prospective validation in contemporary cohorts. If validated, it may help identify high-risk individuals for targeted prevention in the future.

Diabetes mellitus (DM) is a group of metabolic diseases, which is caused by the loss of function of β-cells in the pancreas, which is caused by the autolysis of insulin-producing β-cells, or by their death or de-differentiation as a substitute for insulin resistance (1). DM is a rapidly growing global problem with enormous social, health and economic consequences, and simultaneously being among the most prevalent chronic conditions globally, impacting 828 million individuals aged over 18 years (2).In China, DM has also become a major public health problem that cannot be ignored, with prevalence increasing rapidly from 0.67% in 1980 to 9.7% in 2007, then rising slightly to 11.2% in 2017. In 2019, it is estimated that approximately 824,000 adults will die from diabetes and related complications in China, with its diabetes-related health expenditure estimated ranking second to that of the United States worldwide (3). The “Healthy China Action” serves as China’s core DM control strategy, implementing integrated prevention and treatment through early diagnosis, standardized management, primary care strengthening, health education, and monitoring, integrated with effective screening strategies to identify high-risk groups and mitigate DM incidence and complications (4).

The onset of diabetes is influenced by many factors, such as genes, diet, lifestyle and environment (5) (6). Insulin resistance (IR) is a key mechanism in the development of diabetes (7). Hyperinsulinemic-Euglycemic Clamp (HIEC) is the gold standard reflecting IR (8). For medical screening of the general population, it is clear that the use of HIEC technology for the detection of IR will increase the economic burden (9). Triglyceride glucose-body mass index (TyG-BMI) is calculated from the product of fasting blood glucose (FBG), triglycerides (TG) and body mass index (BMI) d is an indicator of IR (10). As noted by Avagimyan et al., the TyG index is a simple, non-invasive surrogate marker of insulin resistance with proven relevance to cardiovascular outcomes (11). Given that insulin resistance is a common pathophysiological basis for both cardiovascular disease and type 2 diabetes, the TyG index’s predictive value for cardiovascular events indirectly supports its potential utility for diabetes risk assessment. Multiple studies have demonstrated that the TyG index serves as an independent predictor for diabetes risk (12–14). Nevertheless, significant heterogeneity exists in individual metabolic status, particularly regarding BMI as a core indicator of obesity, which is intrinsically linked to insulin sensitivity and diabetes pathogenesis.so TyG-BMI, as a new alternative marker for assessing IR compared to the traditional (15). A prospective large-scale population study conducted in Japan shows that the TyG-BMI has a higher efficiency in reflecting insulin resistance in clinical settings (16).

However, there is still no consistent association between TyG-BMI levels and diabetes (17). In our retrospective cohort study, we attempted to characterize the association of TyG-BMI with incident diabetes in a large Chinese cohort, with the aim of defining a precise relationship between TyG-BMI and incident diabetes.

## Methods

### Data source

The data used in our study came from DRYAD, an online archive that provides the scientific community with free access to a wide range of primary data for collection and download. The platform provided us with a dataset originally provided by Chen (18) et al. Due to the anonymity of the data and the research ethics that have been approved by the Rich Healthcare Group Review Board in previous studies (18), there is no need to reapply for this study. The dataset contains information on 211,833 persons from 11 cities and 32 locations in China. In accordance with DRYAD’s guidelines and terms of service, a secondary analysis of this publicly available dataset was conducted. The dataset is available at https://datadryad.org/dataset/doi:10.5061/dryad.ft8750v.

### Study population

**Figure 1:**
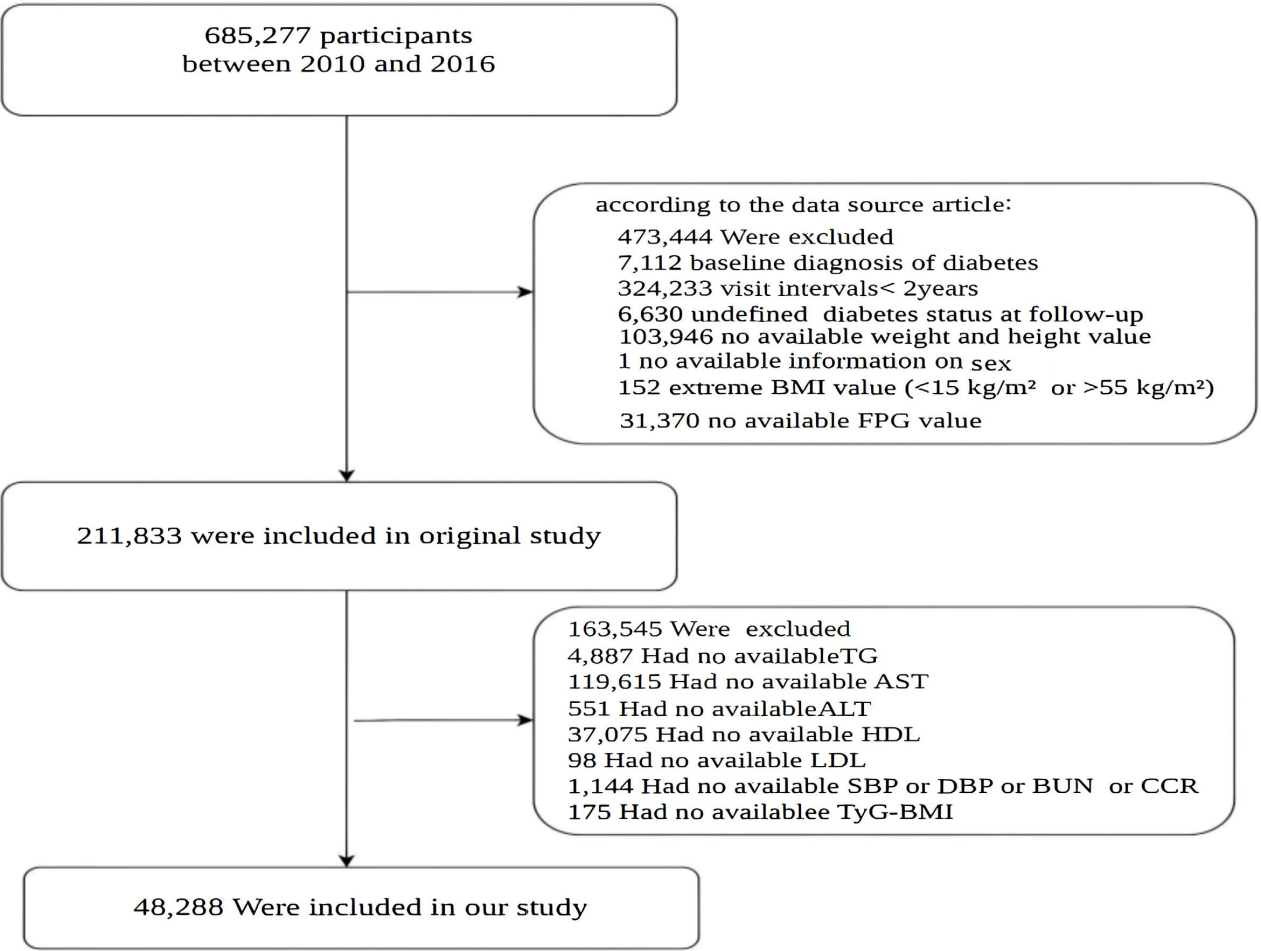
Flow chart of study participants.

The original study recruited a cohort of 685,277 Chinese adults, aged 20, who had attended at least 2 consultations, covering 11 cities and 32 locations in China. This retrospective cohort study excluded participants based on: baseline diagnosis of diabetes; follow-up interval less than 2 years; undefined diabetes status at follow-up; missing weight or height values; missing information on sex; extreme BMI values (<15 or >55 kg/m²); and missing FPG values. the original authors obtained a dataset of 211,833 participants who were free of diabetes at baseline (defined as no self-reported history of diabetes and a fasting plasma glucose <7.00 mmol/L, in accordance with World Health Organization diagnostic criteria (19)).In the present study, participants were excluded for the following reasons:(1) no information on TG levels; (2) no information on aspartate transaminase(AST)and alanine aminotransferase(ALT) levels; (3) no information on high-density lipoprotein cholesterol(HDL) and low-density lipoprotein cholesterol(LDL) levels; (4) no information on systolic blood pressure(SBP), diastolic blood pressure(DBP), blood urea nitrogen(BUN), and creatinine clearance rate (CCR)levels; and (5) no information on TyG-BMI levels. **Figure 1** illustrates the screening process, which resulted in the inclusion of 48,288 participants for secondary analysis.

### Data collection

Data collection and organization were carried out by personnel who had received specific training for these tasks. The initial studies used a uniform environment for obtaining laboratory data and standardized procedures for data processing. Demographic information was collected from these individuals, including age, SBP, DBP, height and weight. Measurements of height and weight were carried out by trained personnel to ensure that the participants were dressed in light clothing without shoes, and the BMI was calculated by weight in kg divided by height (kg/m^2^). The group used a conventional mercury sphygmomanometer to measure blood pressure. Additionally, the expert group evaluated FPG, high-density lipoprotein cholesterol (HDL), blood urea nitrogen (BUN), triglycerides (TG), creatinine clearance rate (CCR), total cholesterol (TC), alanine aminotransferase (ALT), low-density lipoprotein cholesterol (LDL), and aspartate aminotransferase (AST). In each visit to the health check center, participants were requested to complete a detailed questionnaire assessing demographic, lifestyle (including smoking and drinking habits) and family history of chronic disease.

### Calculation and outcome measures

In Chinese clinical practice, FPG and TG are routinely reported in mmol/L; to apply the classical TyG-BMI formula, we converted both values to mg/dL.

TyG-BMI was calculated as *BMI* × *Ln* $\left[TG\left(mg/dL\right)\times FPG\left(mg/dL\right)/2\right]$ (10).

The primary outcome indicator for this study was incident DM defined as an FPG of 7.00 mmol/L or self-reported DM during the follow-up period (19).

### Statistical analysis

Categorical variables were represented as percentages (%), whereas continuous variables were described by mean (standard deviation, SD) or median (interquartile range, IQR), as appropriate. A one-way analysis of variance (normal distribution), Kruskal-Wallis test (skewed distribution), and Chi-Square (categorical variables) were used for comparison between groups. For post-hoc pairwise comparisons following a significant ANOVA, Tukey’s honest significant difference (HSD) test was applied. For the Kruskal-Wallis H test, Dunn’s test with Bonferroni correction was used for multiple comparisons. Cumulative incidence curves were used to estimate the cumulative incidence of diabetes over time across TyG-BMI quartiles. The curves present the cumulative incidence for each quartile group, with 95% confidence bands. Differences between the four curves were compared using the Mantel-Cox log-rank test. In addition, the association of TyG-BMI with diabetes risk was investigated using Cox proportional hazards regression. The Wald test was used to assess the statistical significance of individual covariates in the model. The likelihood ratio test was used as the omnibus test of model coefficients to assess the overall significance of each model. As TyG-BMI is mathematically derived from FPG, TG, and BMI, these components were not included as separate covariates in the regression models to avoid collinearity (**Supplementary Table S1**). For the remaining covariates (age, sex, SBP, DBP, TC, ALT, AST, HDL, LDL, BUN, CCR, family history, smoking, drinking), ALT and AST exhibited moderate correlation (raw GVIF 6.16 and 5.69), but their adjusted GVIF values (2.48 and 2.39) were below the acceptable limit of 3, indicating that collinearity does not compromise model stability. Both variables were retained due to their clinical relevance. Three Cox proportional hazards regression models were constructed. Model 1 was modified for social and demographic characteristics, including age, sex; Model 2 was further modified to measure age, sex, SBP,DBP, TC, HDL, LDL, BUN, CCR, and Model 3 was further adjusted for age, sex, SBP, DBP, TC, ALT, AST, HDL, LDL, BUN, CCR, Diabetes Family History, smoking status, drink status. All the analyses were performed with the statistical software packages R (http://www.R-project.org, The R Foundation) and Free Statistics software versions 2.0. All statistical tests were two-tailed, and a confidence level of 95% was used for all confidence intervals. The standard error of estimation for the hazard ratios was derived from the Cox regression model.

## Results

### Baseline characteristics of participants

In this study, we enrolled 48,288 subjects who were initially DM free. Of these, 1230 had developed diabetes at the time of follow-up, accounting for 2.54%.Participants were categorized into subgroups (Q1-Q4) according to TyG-BMI quartiles using the 25th, 50th, and 75th percentiles (170.28, 194.81, and 221.71, respectively) as cut-offs. The baseline characteristics of the total population are shown in **Table 1**. In the Q4 group, age, proportion of males, BMI, SBP, DBP, ALT, AST, cholesterol, triglycerides, BUN, CCR, and proportion of smokers and drinkers were higher than in the other groups. In the Q4 group, age, proportion of males, BMI, SBP, DBP, FPG, TC, TG, HDL, LDL, ALT, AST, BUN, CCR, and proportion of smokers and drinkers were higher than in the other groups. Pairwise comparisons across TyG-BMI quartiles are shown in continuous variables and categorical variables. For most continuous variables (e.g., age, SBP, DBP, FPG, TG, ALT), all quartiles differed significantly from each other (see letter labels in **Supplementary Figure S1**). For categorical variables, the prevalence of current smoking and drinking increased progressively from Q1 to Q4 (**Supplementary Figure S2**).

**Table 1:** Sociodemographic, laboratory, family history, and lifestyle characteristics of participants according to TyG-BMI quartiles.

Variables	Q1[120.85-170.28)	Q2[170.28-194.81)	Q3[194.81-221.71)	Q4[221.71-340.47]	p
No of subjects	12072	12072	12072	12072	
Age,years	38.7 ± 11.1	43.3 ± 12.6	46.2 ± 13.0	47.5 ± 13.0	< 0.001
Sex, n (%)					< 0.001
Male	3593 (29.8)	6033 (50)	8072 (66.9)	9340 (77.4)	
Female	8479 (70.2)	6039 (50)	4000 (33.1)	2732 (22.6)	
BMI, kg/m^2^	19.7 ± 1.4	22.2 ± 1.2	24.3 ± 1.3	27.5 ± 2.4	< 0.001
SBP, mmHg	111.3 ± 14.0	117.1 ± 15.4	122.2 ± 16.0	127.9 ± 16.7	< 0.001
DBP,mmHg	69.4 ± 9.3	72.5 ± 9.9	76.0 ± 10.5	80.0 ± 11.1	< 0.001
FPG, mmol/L	4.8 ± 0.5	4.9 ± 0.5	5.1 ± 0.6	5.2 ± 0.6	< 0.001
TC, mmol/L	4.5 ± 0.8	4.7 ± 0.8	4.9 ± 0.9	5.1 ± 0.9	< 0.001
TG, mmol/L	0.7 (0.5, 0.9)	0.9 (0.7, 1.2)	1.3 (1.0, 1.7)	2.0 (1.4, 2.7)	< 0.001
HDL, mmol/L	1.5 ± 0.3	1.4 ± 0.3	1.3 ± 0.3	1.3 ± 0.3	< 0.001
LDL, mmol/L	2.5 ± 0.6	2.7 ± 0.6	2.9 ± 0.7	2.9 ± 0.7	< 0.001
ALT, IU/L	13.0 (10.5, 17.6)	16.0 (12.2, 22.5)	20.6 (15.0, 29.0)	28.0 (19.6, 41.2)	< 0.001
AST, IU/L	21.2 ± 9.2	22.9 ± 16.1	24.5 ± 11.3	27.9 ± 11.9	< 0.001
BUN, mmol/L	4.4 ± 1.1	4.6 ± 1.2	4.8 ± 1.2	4.9 ± 1.2	< 0.001
CCR, mmol/L	65.2 ± 14.1	70.5 ± 15.7	74.7 ± 15.3	77.5 ± 16.9	< 0.001
Diabetes Family History,n (%)					0.016
no	11842 (98.1)	11795 (97.7)	11819 (97.9)	11773 (97.5)	
yes	230 (1.9)	277 (2.3)	253 (2.1)	299 (2.5)	
Smoking status,n (%)					< 0.001
Never	2506 (87.9)	2443 (81)	2310 (72.6)	2199 (63)	
Ever smoker	60 (2.1)	113 (3.7)	179 (5.6)	207 (5.9)	
Current smoker	285 (10)	460 (15.3)	691 (21.7)	1082 (31)	
Drink status,n (%)					< 0.001
Never	2497 (87.6)	2395 (79.4)	2365 (74.4)	2370 (67.9)	
Ever drinker	324 (11.4)	567 (18.8)	703 (22.1)	937 (26.9)	
Current drinker	30 (1.1)	54 (1.8)	112 (3.5)	181 (5.2)	

Statistical analysis includes both parametric and non-parametric tests: one-way ANOVA or t-test for normally distributed data, and Kruskal-Wallis test for non-normally distributed data.

Continuous variables were summarized as mean (SD) or medians (quartile interval); categorical variables are presented as n (%). BMI, body mass index; SBP, systolic blood pressure; DBP, diastolic blood pressure; TC, total cholesterol; TG, triglyceride; HDL, high-density lipoprotein cholesterol; LDL, low-density lipoprotein cholesterol; AST, aspartate aminotransferase; ALT, alanine aminotransferase; BUN, blood urea nitrogen; CCR, creatinine clearance rate.

### Relationship between TyG-BMI and the incidence of DM

In this study, the relationship between TyG-BMI and diabetes was analysed using a Cox regression model, and was shown in **Table 2**.In the unadjusted initial model, each 10-unit increase in TyG-BMI was associated with a 25% higher risk of diabetes, with a HR of 1.25 (95% CI 1.23 to 1.26, *P* < 0.001). Partially modified models, which took into account only age and sex, showed a 22% increase in TyG-BMI risk and a HR of 1.22 (95% confidence interval 1.21 to 1.24, p < 0.001). The fully adjusted model showed a 25% increase in risk for diabetes for every 10 units of TyG-BMI, with a 1.25 HR (95% CI 1.20-1.29, p < 0.001). Consistent confidence intervals further support a strong association between TyG-BMI and diabetes progression. Furthermore, we converted TyG-BMI (in 10 units) to a categorical variable and reintroduced it into the model. The HR of the 2nd to 4th quartiles (Q2, Q3, Q4) was 1.45 (0.58-3.67), 5.41 (2.42 - 12.06) and 11.24 (5.05-24.99) in comparison with the first quartile (Q1). This showed that the risk of diabetes in Q2, Q3, and Q4 was 45 percent, 441 percent, and 1024 percent higher. Even after adjusting only for age and sex (Model 1), the HR for Q4 versus Q1 was 11.27 (95% CI: 8.29–15.33, *P*< 0.001), and for Q3 it was 4.45 (3.24–6.12, *P* < 0.001). In Model 2, which further included blood pressure and lipid parameters, the corresponding HRs remained very high (Q3: 4.34, Q4: 10.47, both *P* < 0.001). These findings indicate that the strong predictive value of TyG-BMI for diabetes is robust across all levels of adjustment, even when only age and sex are controlled for. The full results for all covariates in Model 3, including HRs, 95% CIs, and p-values, are presented in **Supplementary Table S2**.

**Table 2:** Relationship between TyG-BMI and the incidence of DM.

Variable	crude.HR (95%CI)	crude.P value	adj.HR (95%CI), P value		
			Model 1	Model 2	Model 3
^a^TyG-BMI index	1.25 (1.23~1.26)	<0.001	1.22 (1.21~1.24),<0.001	1.22 (1.21~1.24),<0.001	1.25 (1.20~1.29),<0.001
^a^TyG-BMI (Quartile)					
Q1	1(Ref)		1(Ref)	1(Ref)	1(Ref)
Q2	2.34(1.65~3.33)	<0.001	1.76 (1.24~2.51),0.002	1.73 (1.21~2.47),<0.001	1.45 (0.58~3.67),0.428
Q3	6.94 (5.07~9.5)	<0.001	4.45 (3.24~6.12),<0.001	4.34 (3.15~6),<0.001	5.41 (2.42~12.06),<0.001
Q4	19.17 (14.19~25.88)	<0.001	11.27 (8.29~15.33),<0.001	10.47 (7.62~14.4),<0.001	11.24 (5.05~24.99),<0.001
P-trend	2.77 (2.58~2.97)	<0.001	2.41 (2.24~2.6),<0.001	2.34 (2.16~2.53),<0.001	2.38 (1.97~2.87),<0.001

TyG-BMI quartiles: Q1 = 120.85-170.27, Q2 = 170.28–194.80, Q3 = 194.81–221.70, Q4 = 221.71-340.47.

Crude model: we did not adjust other covariates.

Model 1 adjusted for age, sex.

Model 2 adjusted for age, sex, SBP, DBP, TC, ALT, AST, HDL, LDL, BUN, CCR.

Model 3 adjusted for age, sex, SBP, DBP, TC, ALT, AST, HDL, LDL, BUN, CCR, Diabetes Family History, smoking status, drink status.

^a^ TyG-BMI as a continuous variable per 10 increase.

Coding for categorical variables: Sex (male = 1, female = 0); Diabetes family history (yes = 1, no = 0); Smoking status (current smoker = 2, ever smoker = 1, never smoker = 0); Drinking status (current drinker = 2, ever drinker = 1, never drinker = 0).

HR, hazard ratio; adj. HR, adjusted hazard ratio.

### Incidence of DM in participants

**Figure 2:**
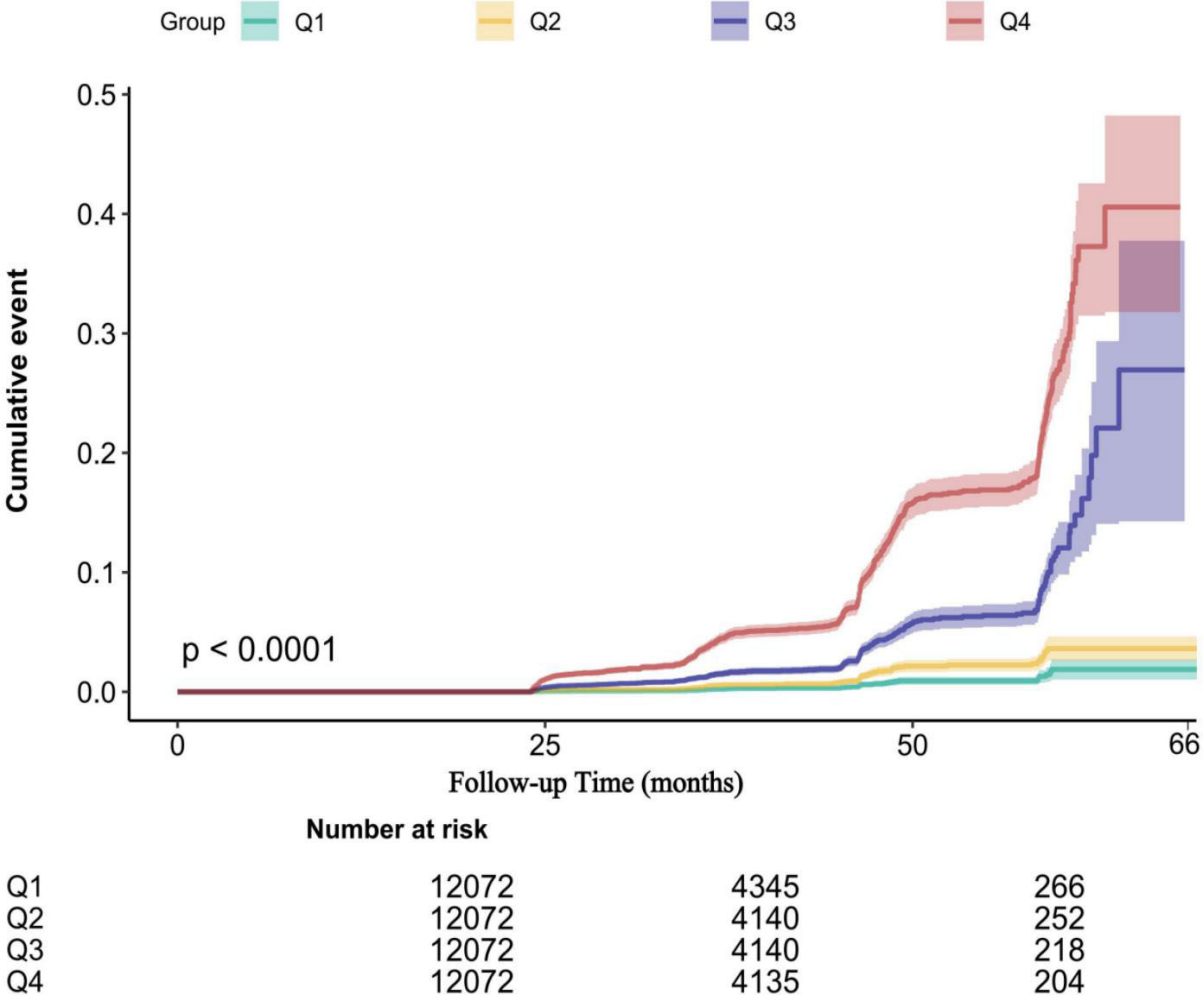
Kaplan-Meier curves for cumulative incidence of diabetes by TyG-BMI quartiles. The solid lines represent the cumulative incidence for each quartile group; shaded areas indicate the 95% confidence intervals. The number at risk is displayed below the x-axis. Differences between curves were compared using the Mantel-Cox log-rank test.

The Kaplan-Meier curves for cumulative incidence of DM are shown in **Figure 2**, which are stratified by TyG-BMI. The cumulative incidence estimates at selected time points, along with their 95% confidence intervals, are provided in **Supplementary Table S3**. The cumulative incidence of DM was significantly different among the four groups (Mantel-Cox log-rank test:χ² = 1024, df = 3, *P* < 0.001). With the increase of TyG-BMI, the cumulative incidence of DM increased, and Q4 (that is, the highest level) was the most likely to have diabetes. Notably, Q3 and Q4 showed a steeper increase over time compared with Q1 and Q2, with Q3 exhibiting a similar pattern to Q4, while Q1 and Q2 remained low and parallel throughout follow-up.

### The non-linear relationship between TyG-BMI and the risk of diabetes in participants

**Figure 3:**
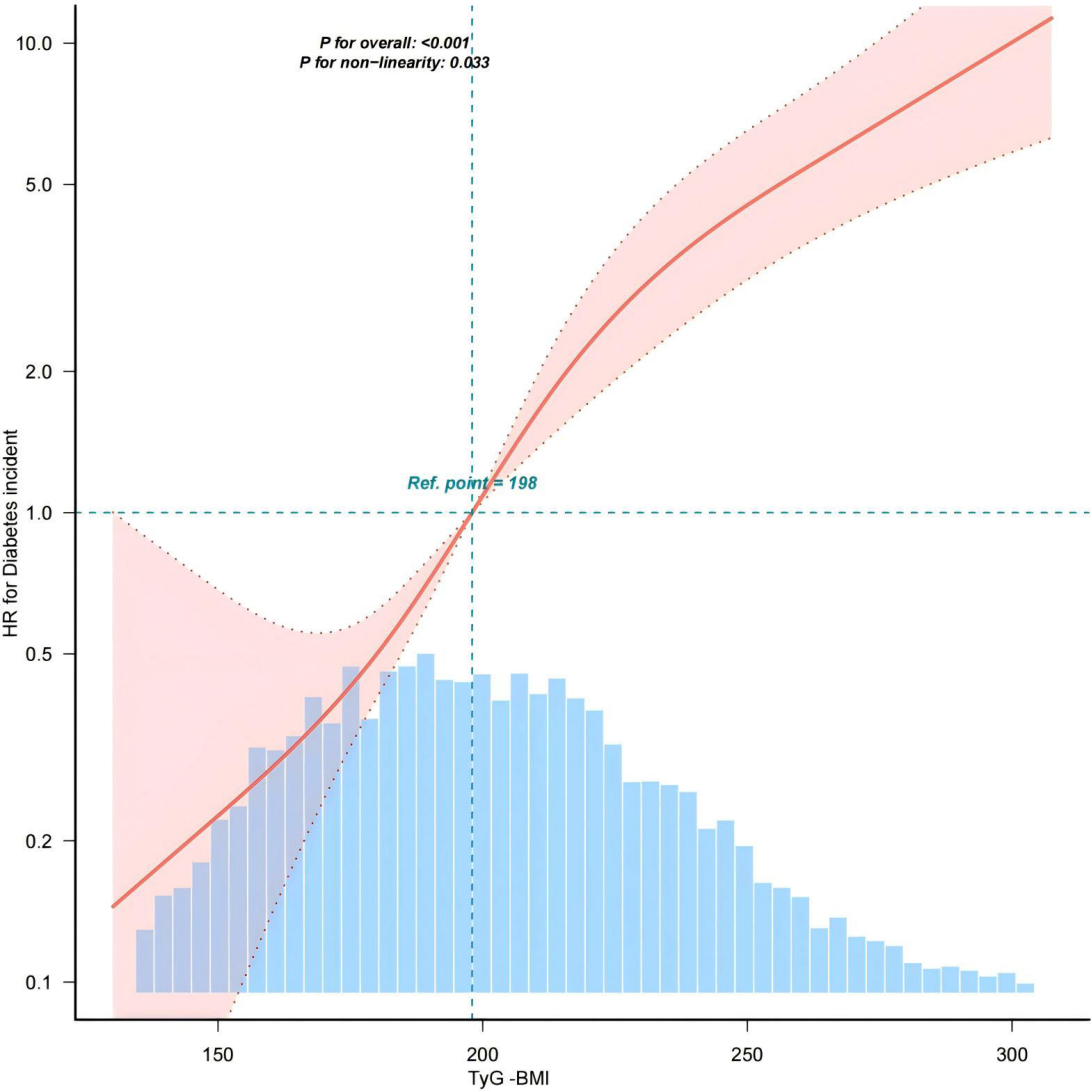
The relationship between the risk of diabetes and the TyG-BMI. The solid line represents the hazard ratio (HR) for incident diabetes across TyG-BMI levels, estimated using a Cox proportional hazards regression model with restricted cubic spline functions (knots placed at the 5th, 35th, 65th, and 95th percentiles of TyG-BMI distribution). The shaded area indicates the 95% confidence interval. Age, sex, SBP, DBP, TC, ALT, AST, BUN, CCR, LDL, HDL, family history of diabetes, alcohol consumption, and smoking status were adjusted.

First, we used a Cox proportional risk regression model with a cubic spline function to assess the relationship between TyG-BMI and the risk of developing diabetes. The results showed a nonlinear relationship between TyG-BMI and the risk of developing DM(**Figure 3**). In the threshold analysis performed, we observed that the odds ratio (OR) for new-onset DM was 1.025 (95% CI: 1.02-1.029, *P* < 0.001) for participants with a TyG-BMI greater than 163.378. This suggests that for every 1 increase in TyG-BMI, the risk of developing diabetes increases by 2.5%.However, when TyG-BMI was <163.38, the association was not statistically significant(HR = 0.928; 95% CI: 0.843–1.022), suggesting that the relationship may be absent or even slightly inverse (**Table 3**).

**Table 3:** Threshold effect analysis of the relationship TyG-BMI of incident DM.

TyG-BMI	HR (95% CI)	P-value
<163.38	0.928 (0.843,1.022)	0.1283
≥163.38	1.025 (1.02,1.029)	<0.001
Likelihood Ratio test	–	0.079

HR, hazard ratio; CI, confidence interval; TyG-BMI, Triglyceride glucose index–body mass index; The likelihood ratio test compared the two-piecewise regression model (with a threshold at 163.38) against a standard linear model.

### Subgroup analysis

**Figure 4:**
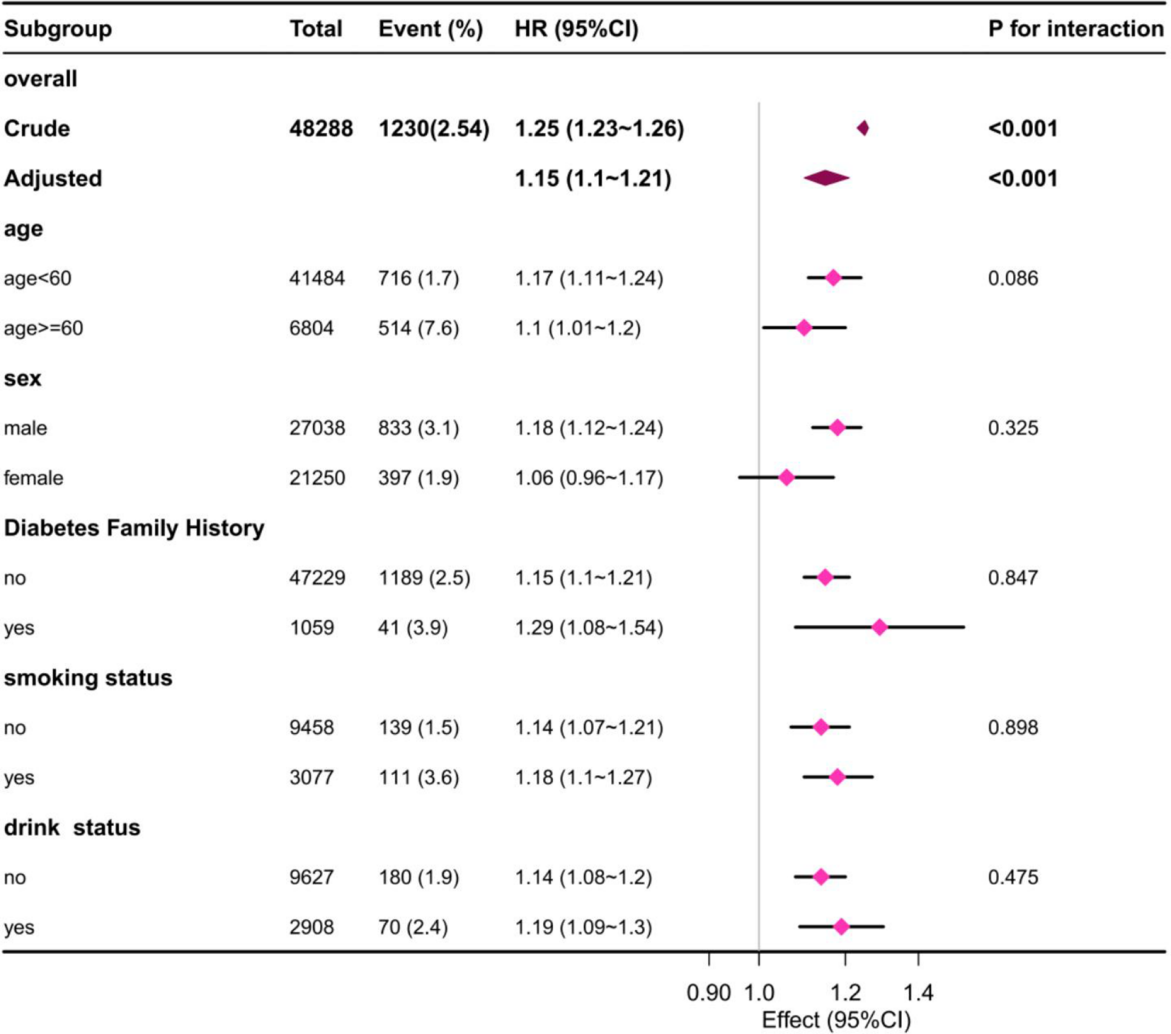
Forrest plot in multivariable logistics analysis between TyG-BMI with DM Hazard ratios (HRs) and 95% confidence intervals (CIs) are shown for each subgroup.

We conducted stratified analyses in several subgroups to examine potential effect modification on the association between TyG-BMI and incident diabetes. The results showed a positive association between TyG-BMI and the development of DM (*P* < 0.001). *T*he associations remained consistent across all subgroups, and no significant interactions were found(*P*>0.05)(**Figure 4**).

## Discussion

In this multicenter retrospective cohort study, we aimed to elucidate the association between TyG-BMI and diabetes risk. As the TyG-BMI index increases, the risk of diabetes shows an increasing trend. Specifically, individuals in the highest two quartiles (Q3 and Q4) had a significantly increased risk of developing diabetes compared to the lowest quartile (Q1) after adequately adjusting for a variety of known confounders, including demographic characteristics, lipids, blood glucose, liver function, renal function, family history, and lifestyle (Model 3: Q3 HR = 5.41, Q4 HR = 11.24). This suggests that extremely high levels of the TyG-BMI index are a strong independent predictor of diabetes incidence, reflecting the significant impact of severe insulin resistance and obesity synergy on diabetes risk. Although the risk in the Q1 group was not significant in the final model, combined with its high risk in the unadjusted model and the significant risk of TyG-BMI as a continuous variable, the upward trend of the TyG-BMI index as a composite indicator should still be considered as a signal of deterioration in metabolic health.

This is similar to the findings of a Chinese national cohort study (20), in which participants with TyG-BMI levels 2, 3, and 4 had DM risk ratios (HRs) of 1.474, 2.250, and 3.142, respectively, compared with those with consistently low TyG-BMI (level 1).Most studies did not continue to explore the specific association between TyG-BMI and the onset of diabetes.

Of particular note is, our findings suggest a threshold effectof TyG-BMI on diabetes incidence. What’s more, our analysis reveals a robust threshold effect at 163.38 in Chinese adults. Below this threshold, the association between TyG-BMI and diabetes risk was not statistically significant, with a wide confidence interval (HR = 0.928; 95% CI: 0.843–1.022). The point estimate suggests a possible protective trend, although this did not reach statistical significance. This finding implies that other factors (e.g., genetic predisposition or environmental triggers) may dominate in low-TyG-BMI individuals, or that the relationship may be absent or even slightly inverse. Beyond the threshold, each 1 increase in TyG-BMI elevates diabetes risk by 2.5%.Our identified threshold (163.38) differs from previous reports. Wang et al. reported an optimal cutoff of 213.3 using ROC analysis in 116,661 participants (21). However, ROC analysis inherently assumes binary classification and maximizes sensitivity and specificity for diagnostic prediction rather than capturing continuous risk trajectories over time, which may explain their higher threshold. Qiao et al. identified an inflection point at 202.9 using restricted cubic splines and reported a J-shaped association (22). Nevertheless, their study was limited to 1,917 participants from Shandong Province, with no adjustment for liver or kidney function parameters, and their reported J-shaped association included a protective phase at low TyG-BMI levels that was not statistically significant. In contrast, our cohort covers 11 cities and 32 locations across China (N = 48,288), with comprehensive adjustments for liver function (ALT, AST), renal function (BUN, CCR), diabetes family history, and lifestyle factors. Notably, our lower threshold (163.38) may be more clinically actionable for early intervention, as it identifies diabetes risk at an earlier stage. The significant likelihood ratio test for the threshold effect (p = 0.002) further supports the robustness of our findings. The TyG-BMI threshold has practical relevance for risk stratification and early, diabetes prevention efforts might prioritize lifestyle modifications (23) (e.g., diet, exercise) over aggressive metabolic monitoring. In contrast, those above the threshold may benefit from intensified surveillance (e.g., annual oral glucose tolerance tests) and targeted therapies (e.g., GLP-1 receptor agonists for concurrent obesity and dysglycemia (24)).However, the clinical applicability of this threshold requires independent validation. From a lipid-centric perspective, the observed threshold effect underscores the threshold-dependent nature of lipotoxicity. Our findings suggest that below the inflection point of 163.38, the body’s compensatory mechanisms may be sufficient to manage the combined metabolic burden of circulating triglycerides and glucose in the context of existing adiposity. However, once this composite load exceeds the threshold, the synergistic pathological effects—likely involving adipose tissue dysfunction, chronic low-grade inflammation, and the direct impairment of insulin signaling by lipid intermediates—may be associated with an increased risk of developing overt diabetes. This supports the concept of a “lipid overload” point, beyond which the risk increases markedly.These measures can effectively help reduce the risk of developing prediabetes.

TG are a major component of lipid metabolism and play a central role in the development of IR. According to a prior study (25), hypertriglyceridemia may lead to the buildup of fatty acids in non-adipose tissues such the heart, muscle, and liver. This could result in ectopic lipid deposition with lipotoxicity, which is recognized as a mechanism for IR. In addition to this, the association between obesity and IR has been well documented. When BMI increases, fat cells become hypertrophic due to saturation of storage capacity. Hypertrophic adipocytes secrete pro-inflammatory factors (e.g. TNF-α, IL-6, MCP-1) and block insulin signaling by inhibiting tyrosine phosphorylation of insulin receptor substrate 1 (IRS-1) through activation of JNK and IKKβ pathways (26).This dual-pathway quantification may help explain TyG-BMI’s superiority over isolated metrics in predicting IR, as it directly mirrors the interplay of lipotoxicity, glucotoxicity, and adiposopathy central to IR development.

Meigs (27) conducted a longitudinal study involving 2,902 participants who were categorized into normal weight, overweight, and obese groups based on BMI. Their findings showed a significant association between higher BMI and an increased risk of developing metabolic syndrome and IR, emphasizing the importance of BMI as a predictor of these conditions. In 2019, Lim et al (12) proposed it as an alternative marker for assessing IR, emphasizing its efficiency when combined with obesity indices such as BMI, waist circumference (WC), and waist-to-height ratio (WHtR).Therefore, TyG indexes combined with obesity indexes can better predict insulin resistance. The TyG-BMI has emerged as a significant marker for assessing IR and related metabolic conditions In a 13-year prospective study of 15,464 diabetes-free adults, Song et al (16) confirmed that TyG-BMI is a robust longitudinal predictor of incident type 2 DM.

This is slightly different from our study. Existing studies not use a Cox proportional risk regression model combined with cubic spline and smoothed curve fitting to examine the nonlinear association between the TyG-BMI and risk of prediabetes, and we identified a critical threshold of 163.38. This observation implies that 163.38could serve as an actionable cut-off for staging cardiometabolic risk: individuals who exceed this value may benefit from intensified lifestyle or pharmacologic intervention, while those under it might be safely monitored within routine primary-care intervals. TyG-BMI as a ready-to-use, low-cost, Accurate screening metric that can identify high risk, still non-diabetic individuals who merit early intervention. Its strong associations with various health outcomes, including hypertension (28), diabetes (29), and liver disease (30), underscore its potential as a valuable tool in both clinical practice and public health initiatives.

This study has several strengths. First of all, we found a non-linear relationship between the level of TyG-BMI and the risk of diabetes in adults. Furthermore, the large sample size of the study, which included a total of 48,288 adults and covered 32 sites and 11 cities in China, reduced the possibility of bias. The inflection point of TyG-BMI was determined by a two-stage Cox proportional risk regression. These findings highlight the critical TyG Index and the risk of diabetes, highlighting the importance of TyG monitoring and early intervention strategies in the prevention of diabetes. A subgroup analysis and an interaction study were also performed. The results indicated that the influence of TyG -BMI on pre-diabetic risk was not significant between subgroups, but there was no significant association between TyG-BMI and factors such as age, sex, alcohol consumption, smoking status, or family history of diabetes. The stability of the results is further verified.

This study has some limitations. First, this is a secondary analysis of a publicly available dataset. As such, our findings are subject to inherent biases present in the original data, including potential selection bias, information bias, and limitations in data collection procedures. Second, confounding factors were not adequately controlled. Although the study adjusted for variables such as age, sex, blood pressure, and lipids, it may have omitted other important confounders (e.g., dietary patterns, physical activity levels, genetic factors, or environmental exposures) that could have influenced the association between TyG-BMI and diabetes risk. Third, the outcome was defined as fasting plasma glucose ≥7.00 mmol/L or self-reported diabetes. Self-reported diabetes status is subject to recall bias, as participants may inaccurately remember or report their diagnosis. Additionally, the study did not utilize a 2-hour oral glucose tolerance test or assess glycated hemoglobin levels, which may lead to an underestimation of diabetes incidence. Fourth, the threshold in this study (163.38) was not validated in an independent cohort (e.g., different ethnic groups, younger adults, or non-Chinese populations), although it was derived from a large sample. Fifth, the data were collected between 2010 and 2016, making the baseline data approximately 10–15 years old. During this period, lifestyle patterns, obesity prevalence, and diabetes diagnostic criteria have substantially changed in China. the generalizability of our findings to the current population may be limited, and the identified threshold (163.38) should be validated in more prospective cohorts. Lastly, the retrospective design while adjustments have been made for a number of confounders, no causal link between TyG-BMI and diabetes risk can be established; we only suggest an association.

Future research efforts should focus on several key priorities: First, prospective validation of the identified 163.38 threshold should be conducted across diverse ethnic populations to establish generalizability. Second, comprehensive integration of TyG-BMI with multi-omics data (including genomics, proteomics, and metabolomics) could elucidate underlying pathophysiological mechanisms. Third, well-designed randomized controlled trials are needed to evaluate the efficacy of TyG-BMI-guided therapeutic interventions in diabetes prevention. Fourth, investigation should be extended to examine potential associations with other metabolic disorders, including non-alcoholic fatty liver disease and cardiovascular conditions. Furthermore, in clinical practice, TyG-BMI helps identify high-risk groups for diabetes (Q3/Q4) and suggests that all people with elevated indices need to pay attention to metabolic health and take appropriate prevention and management measures. Future studies could explore the optimal risk stratification cut-off for TyG-BMI, the development of automated clinical decision-support systems incorporating TyG-BMI monitoring and risk stratification algorithms could facilitate translation of these findings into routine practice. In the future outlook, it is proposed to conduct diagnostic test accuracy studies to evaluate the sensitivity, specificity, and other indicators of TyG-BMI, as well as their inflection points for predicting diabetes, and compare them with other predictive indicators.

## Conclusions

Our findings suggest that TyG-BMI may serve as a surrogate marker for insulin resistance and that a threshold effect exists at approximately 163.38. Nevertheless, this threshold requires prospective validation in contemporary cohorts. If validated in future studies, it could have clinical applications for identifying individuals at increased risk of diabetes and guiding early preventive strategies.

## Data Availability

Publicly available datasets were analyzed in this study.This data can be found here: https://datadryad.org/dataset/doi:10.5061/dryad.ft8750v#citations.
