# Supplementary material for: Non-linear relationship between triglyceride glucose-body mass index and risk of diabetes in adults: a general population-based cohort study of Chinese adults using a publicly available DRYAD dataset
Source: Front Endocrinol (Lausanne). 2026 May 25;17:1823392. doi: 10.3389/fendo.2026.1823392 (PMC13243009; doi:10.3389/fendo.2026.1823392)
Supplement: Supplementary file 1 [file DataSheet1.pdf]

Table S1. Collinearity diagnostics for covariates in the fully adjusted Cox model

Term	GVIF	GVIF^(1/(2*Df))	DF	select
Age	1.382	1.176	1	YES
sex	1.863	1.365	1	YES
SBP	1.982	1.408	1	YES
DBP	1.855	1.362	1	YES
TC	3.346	1.829	1	YES
HDL	1.243	1.115	1	YES
LDL	3.039	1.743	1	YES
ALT	6.158	2.482	1	YES
AST	5.69	2.385	1	YES
BUN	1.131	1.063	1	YES
CCR	1.557	1.248	1	YES
Diabetes Family History	1.055	1.027	1	YES
Smoking status	1.318	1.072	2	YES
Drink status	1.198	1.046	2	YES

Table S2. Multivariable Cox regression results for all covariates in the fully adjusted model (Model 3)

Item	HR(95%CI)	P(Wald's test)	P(LR-test)
Age	1.07 (1.06,1.07)	< 0.001	< 0.001
sex	0.59 (0.52,0.66)	< 0.001	< 0.001
SBP	1.04 (1.04,1.04)	< 0.001	< 0.001
DBP	1.05 (1.04,1.05)	< 0.001	< 0.001
TC	1.32 (1.25,1.4)	< 0.001	< 0.001
HDL	0.83 (0.69,1)	0.048	0.048
LDL	1.3 (1.21,1.41)	< 0.001	< 0.001
ALT	1.0047 (1.0042,1.0053)	< 0.001	< 0.001
AST	1.0063 (1.0054,1.0073)	< 0.001	< 0.001
BUN	1.2 (1.17,1.25)	< 0.001	< 0.001
CCR	1.0057 (1.0043,1.007)	< 0.001	< 0.001
Diabetes Family History(yes vs. no)	1.46 (1.07,1.99)	0.017	0.025
Smoking status (reference: never)			< 0.001
Ever smoker	2.12 (1.33,3.4)	0.002	
Current smoker	2.33 (1.79,3.03)	< 0.001	
Drinking status (reference: never)			0.003
Ever drinker	1.1 (0.81,1.49)	0.553	
Current drinker	2.79 (1.67,4.65)	< 0.001	

Abbreviations: HR, hazard ratio; CI, confidence intervalt.Wald's test p- values are for individual coefficients; LR- test p- values are for the overall contribution of multi- category variables

Table S3. Cumulative incidence of diabetes (95% CI) by TyG-BMI quartile at selected follow-up times

strata	months	n.risk	n.event	n.censor	surv	Cumu	lower	upper
Q1	25.0	9817	1	31	1	0	0.999	1
Q1	30.32	8052	0	4	0.999	0.001	0.999	1
Q1	35.48	6485	1	16	0.998	0.002	0.998	0.999
Q1	40.64	4284	1	9	0.997	0.003	0.996	0.998
Q1	45.10	3859	1	12	0.997	0.003	0.995	0.998
Q1	50.95	1206	0	2	0.991	0.009	0.988	0.994
Q1	55.06	984	0	2	0.991	0.009	0.988	0.994
Q1	60.09	249	0	5	0.981	0.019	0.973	0.99
Q1	65.67	18	0	1	0.981	0.019	0.973	0.99
Q2	25.26	9331	1	37	0.999	0.001	0.998	0.999
Q2	30.42	7701	0	1	0.998	0.002	0.997	0.999
Q2	35.64	6013	1	63	0.996	0.004	0.995	0.997
Q2	40.24	4112	0	7	0.994	0.006	0.992	0.996
Q2	45.60	3431	1	8	0.991	0.009	0.989	0.994
Q2	50.59	1247	0	3	0.978	0.022	0.973	0.984
Q2	55.26	991	0	1	0.978	0.022	0.972	0.983
Q2	60.02	252	0	14	0.964	0.036	0.954	0.974
Q2	65.08	11	0	1	0.964	0.036	0.954	0.974
Q3	25.00	9720	1	38	0.996	0.004	0.995	0.997
Q3	30.39	7868	0	4	0.993	0.007	0.991	0.995
Q3	35.02	6541	1	12	0.989	0.011	0.987	0.991
Q3	40.01	4140	0	1	0.983	0.017	0.98	0.986
Q3	45.01	3719	0	4	0.98	0.020	0.977	0.984
Q3	50.33	1211	1	3	0.94	0.060	0.932	0.949
Q3	55.03	971	0	1	0.936	0.064	0.927	0.946
Q3	60.18	192	0	5	0.88	0.120	0.858	0.902
Q3	65.67	5	0	1	0.731	0.269	0.623	0.857
Q4	25.00	9658	3	27	0.989	0.011	0.988	0.991
Q4	30.06	7832	1	4	0.982	0.018	0.979	0.984
Q4	35.08	6504	0	16	0.97	0.030	0.967	0.974
Q4	40.11	4128	0	3	0.949	0.051	0.943	0.954
Q4	45.37	3396	1	4	0.932	0.068	0.926	0.939
Q4	50.00	1246	1	3	0.842	0.158	0.829	0.855
Q4	55.99	947	0	3	0.831	0.170	0.817	0.845
Q4	60.22	175	1	6	0.719	0.281	0.691	0.749
Q4	65.97	4	0	1	0.594	0.409	0.518	0.682

Note: Time points are approximate; exact times are given in the full survival table. Cumulative incidence = 1 – Kaplan-Meier survival probability. CI = confidence interval.

Figure S1 Baseline continuous variables across TyG-BMI quartiles with pairwise comparison results

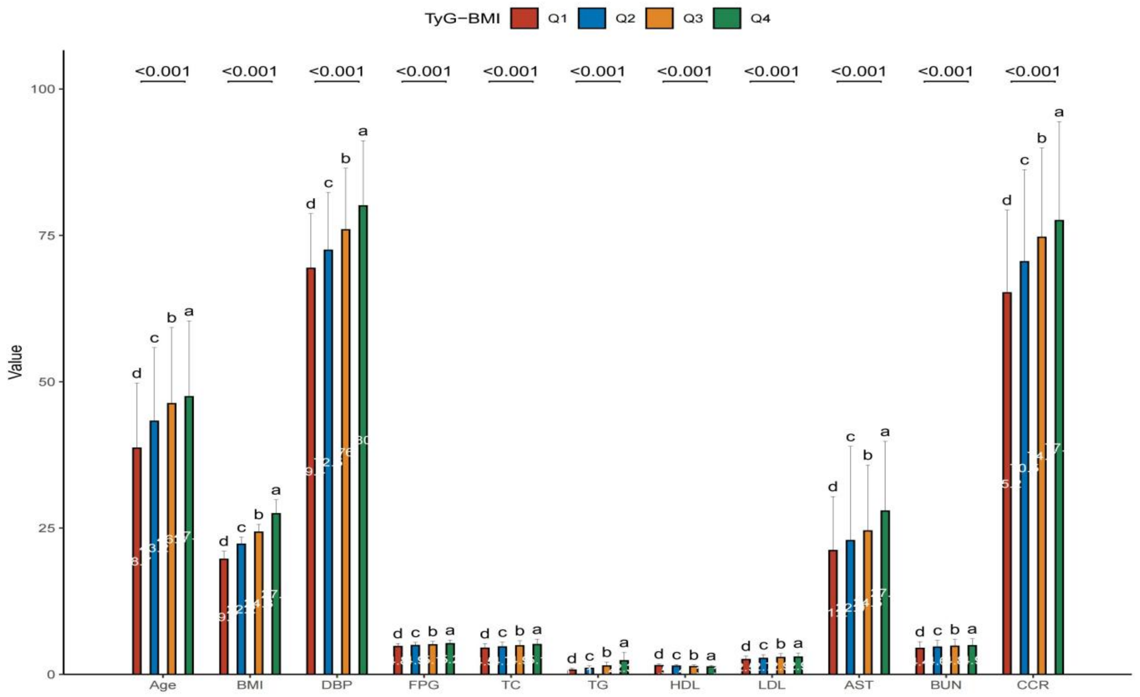

Figure S1 Baseline continuous variables across TyG-BMI quartiles with pairwise comparison results.The bar chart shows the mean (or median) values of age, BMI, DBP, FPG, TC, TG, HDL, LDL, AST, BUN, and CCR for each TyG-BMI quartile (Q1 to Q4). Different superscript letters (a, b, c, d) above the bars indicate statistically significant differences between quartiles ($p < 0.05$) based on one-way ANOVA with Tukey's HSD post-hoc test for normally distributed variables, or Kruskal-Wallis test with Dunn's test and Bonferroni correction for non-normally distributed variables.

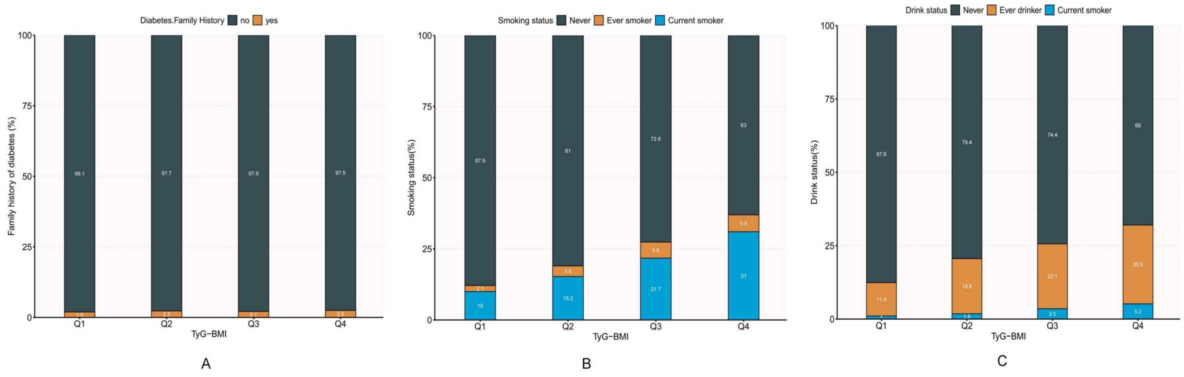

Figure S2. Distribution of categorical variables across TyG-BMI quartiles.The bar chart shows the relative percentages of family history, smoking status, and drinking status categories

across TyG-BMI quartiles (Q1 to Q4). Values are expressed as a percentage of the maximum observed value (set to 100%).
